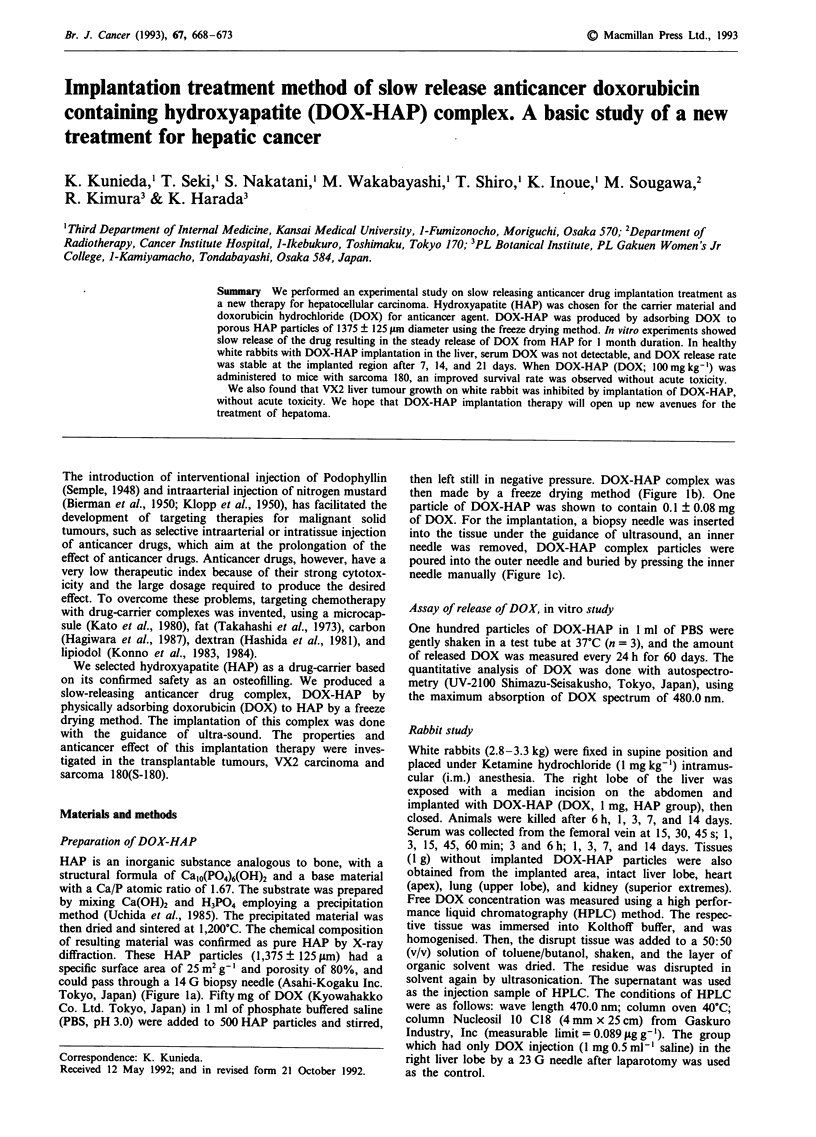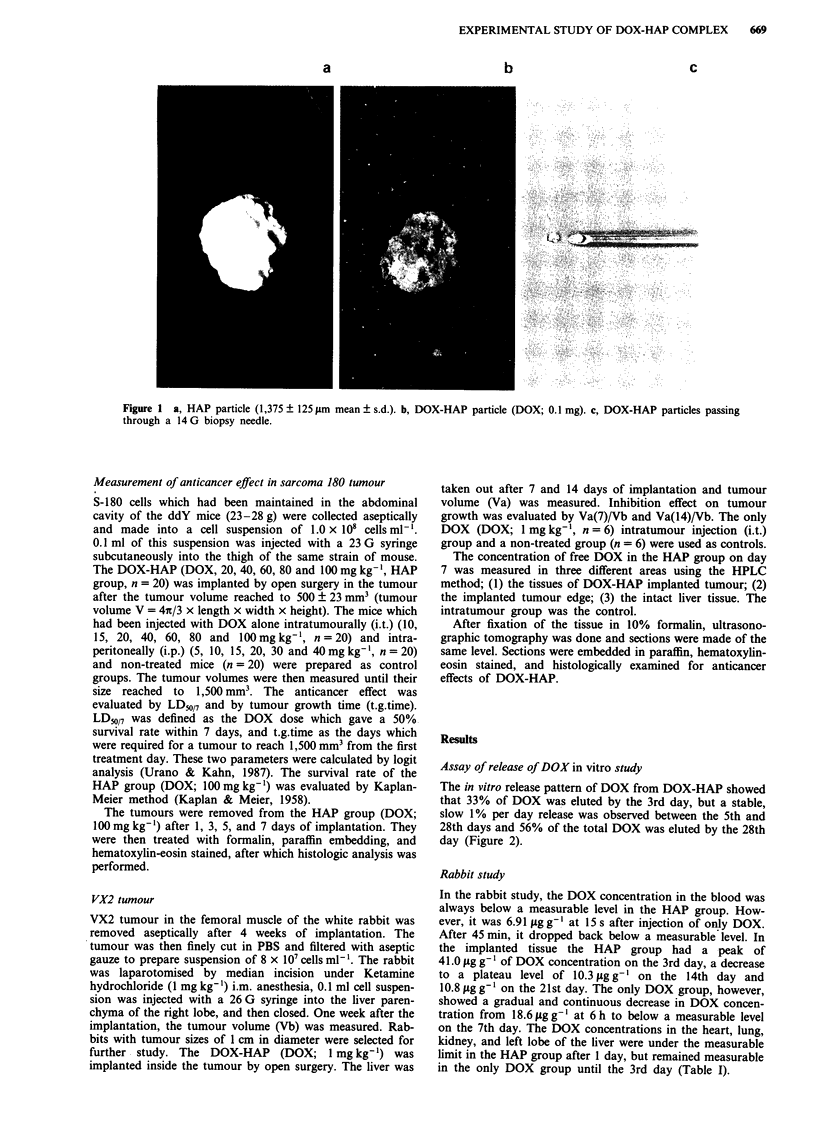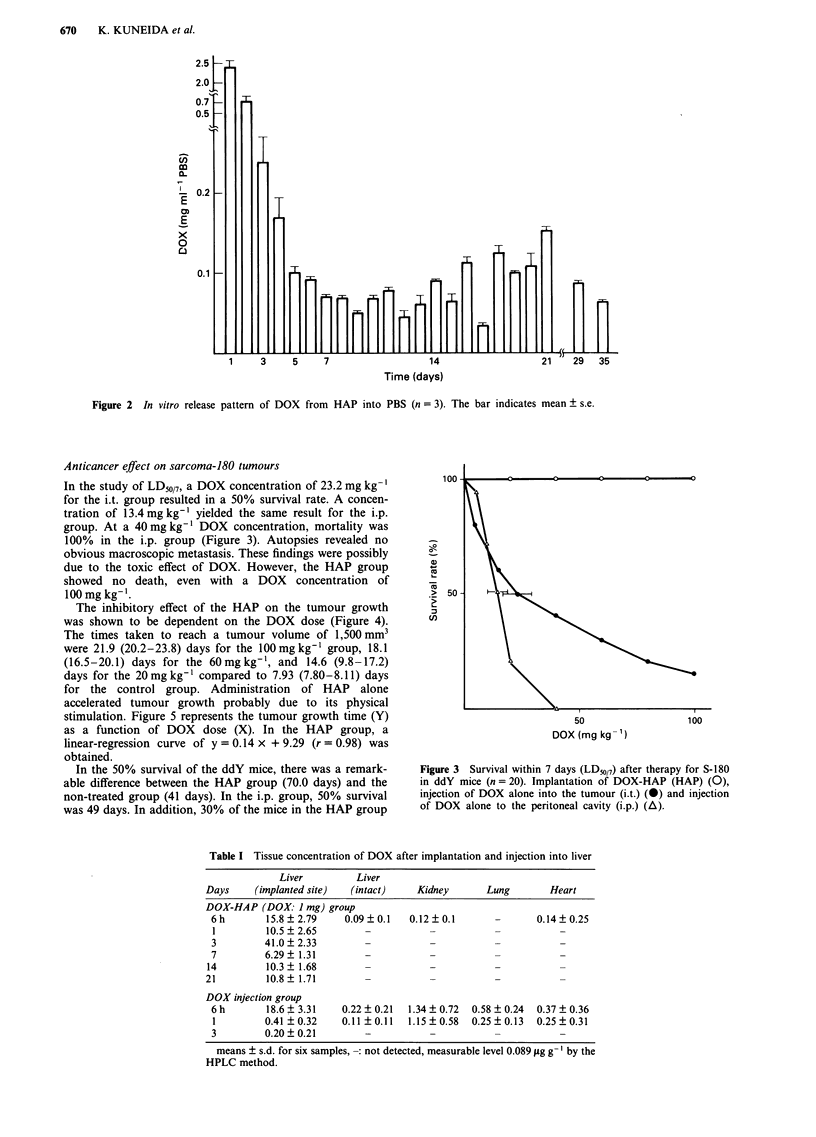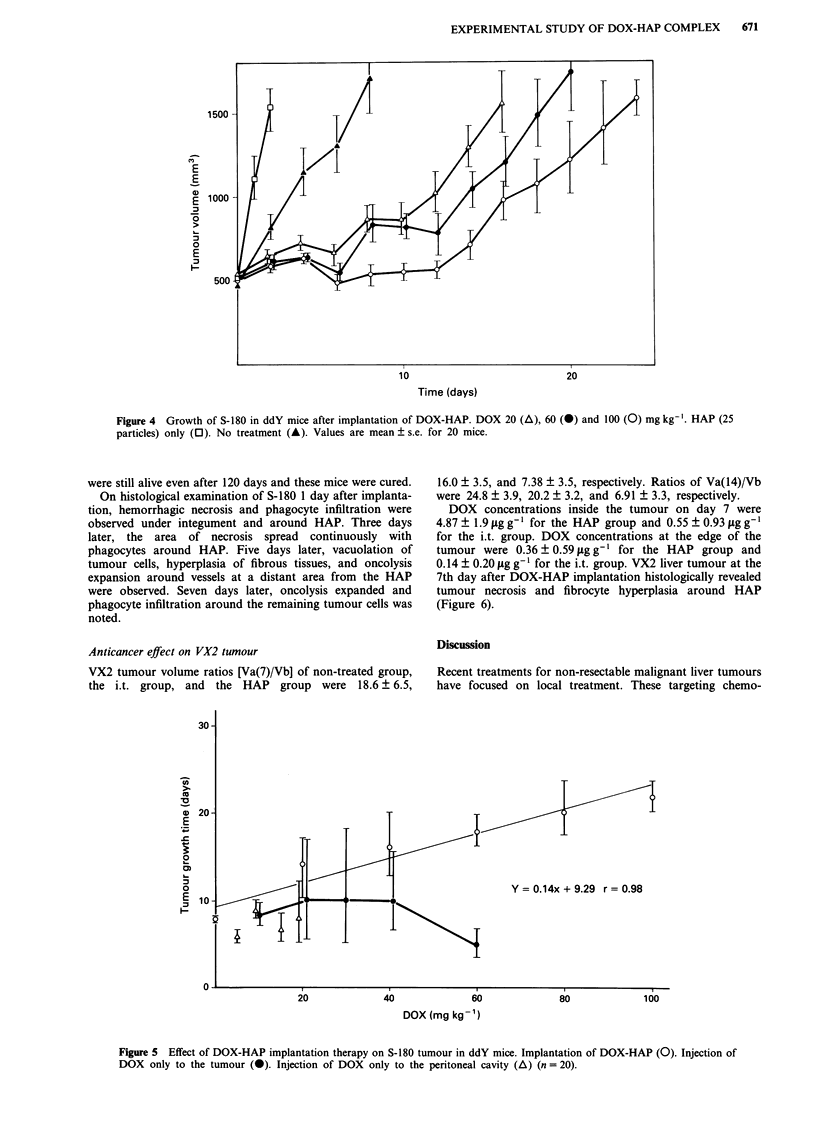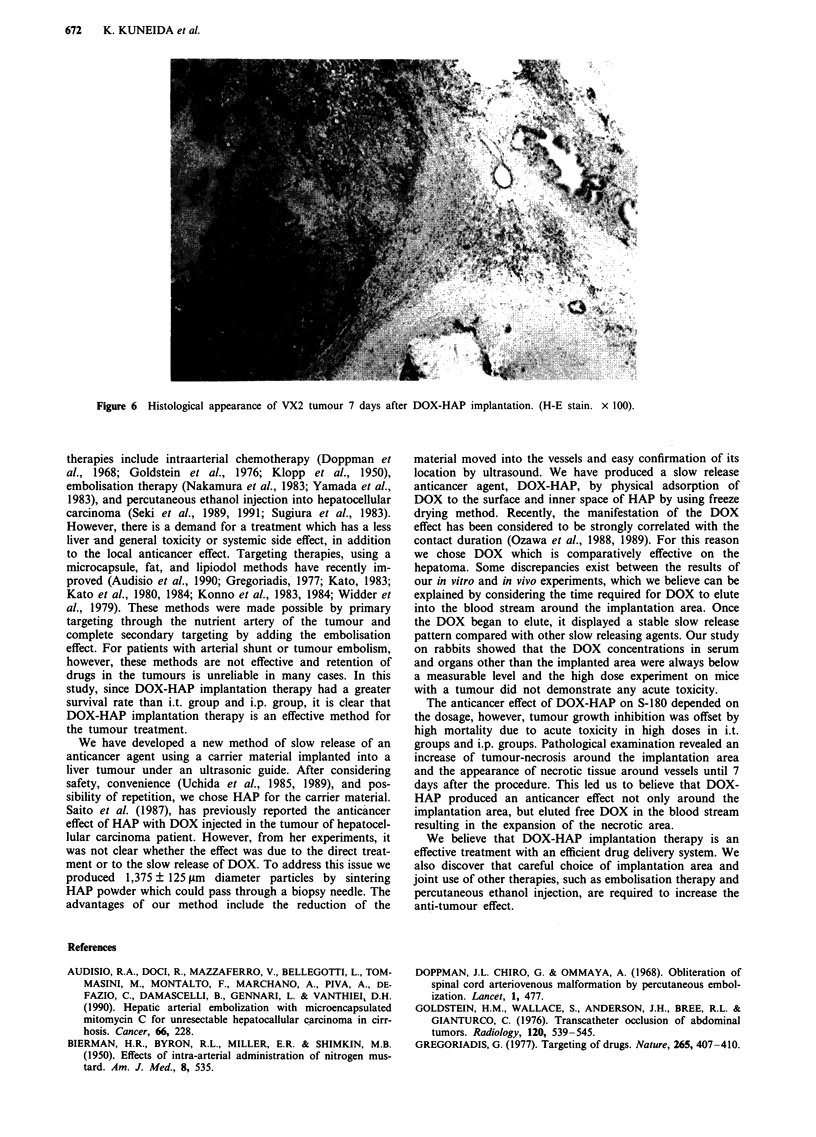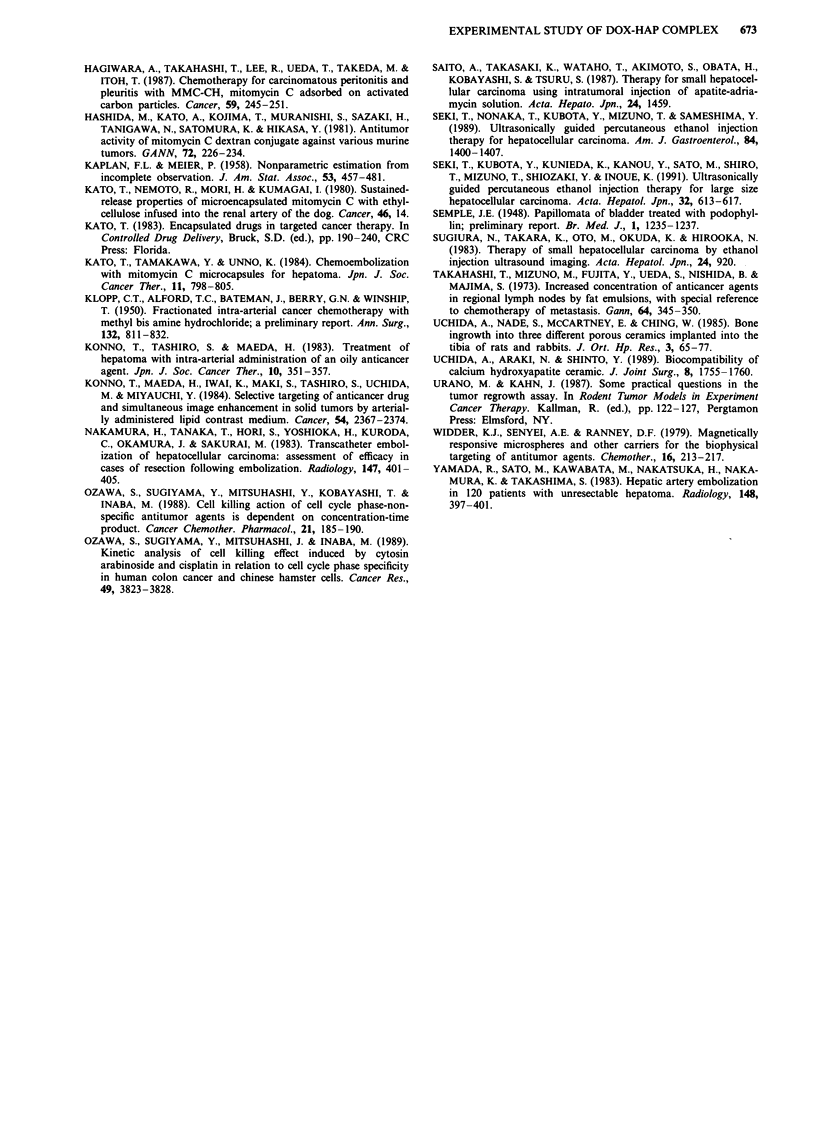# Implantation treatment method of slow release anticancer doxorubicin containing hydroxyapatite (DOX-HAP) complex. A basic study of a new treatment for hepatic cancer.

**DOI:** 10.1038/bjc.1993.124

**Published:** 1993-04

**Authors:** K. Kunieda, T. Seki, S. Nakatani, M. Wakabayashi, T. Shiro, K. Inoue, M. Sougawa, R. Kimura, K. Harada

**Affiliations:** Third Department of Internal Medicine, Kansai Medical University, Osaka, Japan.

## Abstract

**Images:**


Br. J. Cancer (1993), 67, 668-673 © Macmillan Press Ltd., 1993

### Implantation treatment method of slow release anticancer doxorubicin containing hydroxyapatite (DOX-HAP) complex. A basic study of a new treatment for hepatic cancer

K. Kunieda,[1] T. Seki,[1] S. Nakatani,[1] M. Wakabayashi,[1] T. Shiro,[1] K. Inoue,[1] M. Sougawa,[2] R. Kimura[3] & K. Harada[3]

[1]*Third Department of Internal Medicine, Kansai Medical University, 1-Fumizonocho, Moriguchi, Osaka 570;* [2]*Department of Radiotherapy, Cancer Institute Hospital, 1-Ikebukuro, Toshimaku, Tokyo 170;* [3]*PL Botanical Institute, PL Gakuen Women's Jr College, 1-Kamiyamacho, Tondabayashi, Osaka 584, Japan.*

**Summary** We performed an experimental study on slow releasing anticancer drug implantation treatment as a new therapy for hepatocellular carcinoma. Hydroxyapatite (HAP) was chosen for the carrier material and doxorubicin hydrochloride (DOX) for anticancer agent. DOX-HAP was produced by adsorbing DOX to porous HAP particles of 1375 ± 125 μm diameter using the freeze drying method. *In vitro* experiments showed slow release of the drug resulting in the steady release of DOX from HAP for 1 month duration. In healthy white rabbits with DOX-HAP implantation in the liver, serum DOX was not detectable, and DOX release rate was stable at the implanted region after 7, 14, and 21 days. When DOX-HAP (DOX; 100 mg kg$^{-1}$) was administered to mice with sarcoma 180, an improved survival rate was observed without acute toxicity.

We also found that VX2 liver tumour growth on white rabbit was inhibited by implantation of DOX-HAP, without acute toxicity. We hope that DOX-HAP implantation therapy will open up new avenues for the treatment of hepatoma.

The introduction of interventional injection of Podophyllin (Semple, 1948) and intraarterial injection of nitrogen mustard (Bierman *et al.*, 1950; Klopp *et al.*, 1950), has facilitated the development of targeting therapies for malignant solid tumours, such as selective intraarterial or intratissue injection of anticancer drugs, which aim at the prolongation of the effect of anticancer drugs. Anticancer drugs, however, have a very low therapeutic index because of their strong cytotoxicity and the large dosage required to produce the desired effect. To overcome these problems, targeting chemotherapy with drug-carrier complexes was invented, using a microcapsule (Kato *et al.*, 1980), fat (Takahashi *et al.*, 1973), carbon (Hagiwara *et al.*, 1987), dextran (Hashida *et al.*, 1981), and lipiodol (Konno *et al.*, 1983, 1984).

We selected hydroxyapatite (HAP) as a drug-carrier based on its confirmed safety as an osteofilling. We produced a slow-releasing anticancer drug complex, DOX-HAP by physically adsorbing doxorubicin (DOX) to HAP by a freeze drying method. The implantation of this complex was done with the guidance of ultra-sound. The properties and anticancer effect of this implantation therapy were investigated in the transplantable tumours, VX2 carcinoma and sarcoma 180(S-180).

### Materials and methods

### *Preparation of DOX-HAP*

HAP is an inorganic substance analogous to bone, with a structural formula of $Ca_{10}(PO_4)_6(OH)_2$ and a base material with a Ca/P atomic ratio of 1.67. The substrate was prepared by mixing $Ca(OH)_2$ and $H_3PO_4$ employing a precipitation method (Uchida *et al.*, 1985). The precipitated material was then dried and sintered at 1,200°C. The chemical composition of resulting material was confirmed as pure HAP by X-ray diffraction. These HAP particles (1,375 ± 125 μm) had a specific surface area of 25 m$^2$ g$^{-1}$ and porosity of 80%, and could pass through a 14 G biopsy needle (Asahi-Kogaku Inc. Tokyo, Japan) (Figure 1a). Fifty mg of DOX (Kyowahakko Co. Ltd. Tokyo, Japan) in 1 ml of phosphate buffered saline (PBS, pH 3.0) were added to 500 HAP particles and stirred, then left still in negative pressure. DOX-HAP complex was then made by a freeze drying method (Figure 1b). One particle of DOX-HAP was shown to contain 0.1 ± 0.08 mg of DOX. For the implantation, a biopsy needle was inserted into the tissue under the guidance of ultrasound, an inner needle was removed, DOX-HAP complex particles were poured into the outer needle and buried by pressing the inner needle manually (Figure 1c).

### *Assay of release of DOX,* in vitro *study*

One hundred particles of DOX-HAP in 1 ml of PBS were gently shaken in a test tube at 37°C ($n$ = 3), and the amount of released DOX was measured every 24 h for 60 days. The quantitative analysis of DOX was done with autospectrometry (UV-2100 Shimazu-Seisakusho, Tokyo, Japan), using the maximum absorption of DOX spectrum of 480.0 nm.

### *Rabbit study*

White rabbits (2.8-3.3 kg) were fixed in supine position and placed under Ketamine hydrochloride (1 mg kg$^{-1}$) intramuscular (i.m.) anesthesia. The right lobe of the liver was exposed with a median incision on the abdomen and implanted with DOX-HAP (DOX, 1 mg, HAP group), then closed. Animals were killed after 6 h, 1, 3, 7, and 14 days. Serum was collected from the femoral vein at 15, 30, 45 s; 1, 3, 15, 45, 60 min; 3 and 6 h; 1, 3, 7, and 14 days. Tissues (1 g) without implanted DOX-HAP particles were also obtained from the implanted area, intact liver lobe, heart (apex), lung (upper lobe), and kidney (superior extremes). Free DOX concentration was measured using a high performance liquid chromatography (HPLC) method. The respective tissue was immersed into Kolthoff buffer, and was homogenised. Then, the disrupt tissue was added to a 50:50 (v/v) solution of toluene/butanol, shaken, and the layer of organic solvent was dried. The residue was disrupted in solvent again by ultrasonication. The supernatant was used as the injection sample of HPLC. The conditions of HPLC were as follows: wave length 470.0 nm; column oven 40°C; column Nucleosil 10 C18 (4 mm × 25 cm) from Gaskuro Industry, Inc (measurable limit = 0.089 μg g$^{-1}$). The group which had only DOX injection (1 mg 0.5 ml$^{-1}$ saline) in the right liver lobe by a 23 G needle after laparotomy was used as the control.

Correspondence: K. Kunieda.
Received 12 May 1992; and in revised form 21 October 1992.

**Figure 1** **a**, HAP particle (1,375 ± 125 μm mean ± s.d.). **b**, DOX-HAP particle (DOX; 0.1 mg). **c**, DOX-HAP particles passing through a 14 G biopsy needle.

*Measurement of anticancer effect in sarcoma 180 tumour*

S-180 cells which had been maintained in the abdominal cavity of the ddY mice (23–28 g) were collected aseptically and made into a cell suspension of $1.0 \times 10^8$ cells $ml^{-1}$. 0.1 ml of this suspension was injected with a 23 G syringe subcutaneously into the thigh of the same strain of mouse. The DOX-HAP (DOX, 20, 40, 60, 80 and 100 mg $kg^{-1}$, HAP group, $n = 20$) was implanted by open surgery in the tumour after the tumour volume reached to 500 ± 23 $mm^3$ (tumour volume $V = 4\pi/3 \times$ length $\times$ width $\times$ height). The mice which had been injected with DOX alone intratumourally (i.t.) (10, 15, 20, 40, 60, 80 and 100 mg $kg^{-1}$, $n = 20$) and intraperitoneally (i.p.) (5, 10, 15, 20, 30 and 40 mg $kg^{-1}$, $n = 20$) and non-treated mice ($n = 20$) were prepared as control groups. The tumour volumes were then measured until their size reached to 1,500 $mm^3$. The anticancer effect was evaluated by $LD_{50/7}$ and by tumour growth time (t.g.time). $LD_{50/7}$ was defined as the DOX dose which gave a 50% survival rate within 7 days, and t.g.time as the days which were required for a tumour to reach 1,500 $mm^3$ from the first treatment day. These two parameters were calculated by logit analysis (Urano & Kahn, 1987). The survival rate of the HAP group (DOX; 100 mg $kg^{-1}$) was evaluated by Kaplan-Meier method (Kaplan & Meier, 1958).

The tumours were removed from the HAP group (DOX; 100 mg $kg^{-1}$) after 1, 3, 5, and 7 days of implantation. They were then treated with formalin, paraffin embedding, and hematoxylin-eosin stained, after which histologic analysis was performed.

*VX2 tumour*

VX2 tumour in the femoral muscle of the white rabbit was removed aseptically after 4 weeks of implantation. The tumour was then finely cut in PBS and filtered with aseptic gauze to prepare suspension of $8 \times 10^7$ cells $ml^{-1}$. The rabbit was laparotomised by median incision under Ketamine hydrochloride (1 mg $kg^{-1}$) i.m. anesthesia, 0.1 ml cell suspension was injected with a 26 G syringe into the liver parenchyma of the right lobe, and then closed. One week after the implantation, the tumour volume (Vb) was measured. Rabbits with tumour sizes of 1 cm in diameter were selected for further study. The DOX-HAP (DOX; 1 mg $kg^{-1}$) was implanted inside the tumour by open surgery. The liver was taken out after 7 and 14 days of implantation and tumour volume (Va) was measured. Inhibition effect on tumour growth was evaluated by Va(7)/Vb and Va(14)/Vb. The only DOX (DOX; 1 mg $kg^{-1}$, $n = 6$) intratumour injection (i.t.) group and a non-treated group ($n = 6$) were used as controls.

The concentration of free DOX in the HAP group on day 7 was measured in three different areas using the HPLC method; (1) the tissues of DOX-HAP implanted tumour; (2) the implanted tumour edge; (3) the intact liver tissue. The intratumour group was the control.

After fixation of the tissue in 10% formalin, ultrasonographic tomography was done and sections were made of the same level. Sections were embedded in paraffin, hematoxylin-eosin stained, and histologically examined for anticancer effects of DOX-HAP.

### Results

*Assay of release of DOX* in vitro *study*

The *in vitro* release pattern of DOX from DOX-HAP showed that 33% of DOX was eluted by the 3rd day, but a stable, slow 1% per day release was observed between the 5th and 28th days and 56% of the total DOX was eluted by the 28th day (Figure 2).

*Rabbit study*

In the rabbit study, the DOX concentration in the blood was always below a measurable level in the HAP group. However, it was 6.91 μg $g^{-1}$ at 15 s after injection of only DOX. After 45 min, it dropped back below a measurable level. In the implanted tissue the HAP group had a peak of 41.0 μg $g^{-1}$ of DOX concentration on the 3rd day, a decrease to a plateau level of 10.3 μg $g^{-1}$ on the 14th day and 10.8 μg $g^{-1}$ on the 21st day. The only DOX group, however, showed a gradual and continuous decrease in DOX concentration from 18.6 μg $g^{-1}$ at 6 h to below a measurable level on the 7th day. The DOX concentrations in the heart, lung, kidney, and left lobe of the liver were under the measurable limit in the HAP group after 1 day, but remained measurable in the only DOX group until the 3rd day (Table I).

**Figure 2** *In vitro* release pattern of DOX from HAP into PBS ($n = 3$). The bar indicates mean ± s.e.

*Anticancer effect on sarcoma-180 tumours*

In the study of $LD_{50/7}$, a DOX concentration of 23.2 mg $kg^{-1}$ for the i.t. group resulted in a 50% survival rate. A concentration of 13.4 mg $kg^{-1}$ yielded the same result for the i.p. group. At a 40 mg $kg^{-1}$ DOX concentration, mortality was 100% in the i.p. group (Figure 3). Autopsies revealed no obvious macroscopic metastasis. These findings were possibly due to the toxic effect of DOX. However, the HAP group showed no death, even with a DOX concentration of 100 mg $kg^{-1}$.

The inhibitory effect of the HAP on the tumour growth was shown to be dependent on the DOX dose (Figure 4). The times taken to reach a tumour volume of 1,500 $mm^3$ were 21.9 (20.2–23.8) days for the 100 mg $kg^{-1}$ group, 18.1 (16.5–20.1) days for the 60 mg $kg^{-1}$, and 14.6 (9.8–17.2) days for the 20 mg $kg^{-1}$ compared to 7.93 (7.80–8.11) days for the control group. Administration of HAP alone accelerated tumour growth probably due to its physical stimulation. Figure 5 represents the tumour growth time (Y) as a function of DOX dose (X). In the HAP group, a linear-regression curve of $y = 0.14 \times + 9.29$ ($r = 0.98$) was obtained.

In the 50% survival of the ddY mice, there was a remarkable difference between the HAP group (70.0 days) and the non-treated group (41 days). In the i.p. group, 50% survival was 49 days. In addition, 30% of the mice in the HAP group

**Figure 3** Survival within 7 days ($LD_{50/7}$) after therapy for S-180 in ddY mice ($n = 20$). Implantation of DOX-HAP (HAP) (○), injection of DOX alone into the tumour (i.t.) (●) and injection of DOX alone to the peritoneal cavity (i.p.) (△).

**Table I** Tissue concentration of DOX after implantation and injection into liver

| Days | *Liver (implanted site)* | *Liver (intact)* | *Kidney* | *Lung* | *Heart* |
|---|---|---|---|---|---|
| *DOX-HAP (DOX: 1 mg) group* | | | | | |
| 6 h | 15.8 ± 2.79 | 0.09 ± 0.1 | 0.12 ± 0.1 | – | 0.14 ± 0.25 |
| 1 | 10.5 ± 2.65 | – | – | – | – |
| 3 | 41.0 ± 2.33 | – | – | – | – |
| 7 | 6.29 ± 1.31 | – | – | – | – |
| 14 | 10.3 ± 1.68 | – | – | – | – |
| 21 | 10.8 ± 1.71 | – | – | – | – |
| *DOX injection group* | | | | | |
| 6 h | 18.6 ± 3.31 | 0.22 ± 0.21 | 1.34 ± 0.72 | 0.58 ± 0.24 | 0.37 ± 0.36 |
| 1 | 0.41 ± 0.32 | 0.11 ± 0.11 | 1.15 ± 0.58 | 0.25 ± 0.13 | 0.25 ± 0.31 |
| 3 | 0.20 ± 0.21 | – | – | – | – |

means ± s.d. for six samples, –: not detected, measurable level 0.089 μg $g^{-1}$ by the HPLC method.

**Figure 4** Growth of S-180 in ddY mice after implantation of DOX-HAP. DOX 20 (△), 60 (●) and 100 (○) mg $kg^{-1}$. HAP (25 particles) only (□). No treatment (▲). Values are mean ± s.e. for 20 mice.

were still alive even after 120 days and these mice were cured.

On histological examination of S-180 1 day after implantation, hemorrhagic necrosis and phagocyte infiltration were observed under integument and around HAP. Three days later, the area of necrosis spread continuously with phagocytes around HAP. Five days later, vacuolation of tumour cells, hyperplasia of fibrous tissues, and oncolysis expansion around vessels at a distant area from the HAP were observed. Seven days later, oncolysis expanded and phagocyte infiltration around the remaining tumour cells was noted.

*Anticancer effect on VX2 tumour*

VX2 tumour volume ratios [Va(7)/Vb] of non-treated group, the i.t. group, and the HAP group were 18.6 ± 6.5, 16.0 ± 3.5, and 7.38 ± 3.5, respectively. Ratios of Va(14)/Vb were 24.8 ± 3.9, 20.2 ± 3.2, and 6.91 ± 3.3, respectively.

DOX concentrations inside the tumour on day 7 were 4.87 ± 1.9 μg $g^{-1}$ for the HAP group and 0.55 ± 0.93 μg $g^{-1}$ for the i.t. group. DOX concentrations at the edge of the tumour were 0.36 ± 0.59 μg $g^{-1}$ for the HAP group and 0.14 ± 0.20 μg $g^{-1}$ for the i.t. group. VX2 liver tumour at the 7th day after DOX-HAP implantation histologically revealed tumour necrosis and fibrocyte hyperplasia around HAP (Figure 6).

### Discussion

Recent treatments for non-resectable malignant liver tumours have focused on local treatment. These targeting chemo-

**Figure 5** Effect of DOX-HAP implantation therapy on S-180 tumour in ddY mice. Implantation of DOX-HAP (○). Injection of DOX only to the tumour (●). Injection of DOX only to the peritoneal cavity (△) ($n = 20$).

**Figure 6** Histological appearance of VX2 tumour 7 days after DOX-HAP implantation. (H-E stain. × 100).

therapies include intraarterial chemotherapy (Doppman *et al.*, 1968; Goldstein *et al.*, 1976; Klopp *et al.*, 1950), embolisation therapy (Nakamura *et al.*, 1983; Yamada *et al.*, 1983), and percutaneous ethanol injection into hepatocellular carcinoma (Seki *et al.*, 1989, 1991; Sugiura *et al.*, 1983). However, there is a demand for a treatment which has a less liver and general toxicity or systemic side effect, in addition to the local anticancer effect. Targeting therapies, using a microcapsule, fat, and lipiodol methods have recently improved (Audisio *et al.*, 1990; Gregoriadis, 1977; Kato, 1983; Kato *et al.*, 1980, 1984; Konno *et al.*, 1983, 1984; Widder *et al.*, 1979). These methods were made possible by primary targeting through the nutrient artery of the tumour and complete secondary targeting by adding the embolisation effect. For patients with arterial shunt or tumour embolism, however, these methods are not effective and retention of drugs in the tumours is unreliable in many cases. In this study, since DOX-HAP implantation therapy had a greater survival rate than i.t. group and i.p. group, it is clear that DOX-HAP implantation therapy is an effective method for the tumour treatment.

We have developed a new method of slow release of an anticancer agent using a carrier material implanted into a liver tumour under an ultrasonic guide. After considering safety, convenience (Uchida *et al.*, 1985, 1989), and possibility of repetition, we chose HAP for the carrier material. Saito *et al.* (1987), has previously reported the anticancer effect of HAP with DOX injected in the tumour of hepatocellular carcinoma patient. However, from her experiments, it was not clear whether the effect was due to the direct treatment or to the slow release of DOX. To address this issue we produced $1,375 \pm 125\,\mu m$ diameter particles by sintering HAP powder which could pass through a biopsy needle. The advantages of our method include the reduction of the material moved into the vessels and easy confirmation of its location by ultrasound. We have produced a slow release anticancer agent, DOX-HAP, by physical adsorption of DOX to the surface and inner space of HAP by using freeze drying method. Recently, the manifestation of the DOX effect has been considered to be strongly correlated with the contact duration (Ozawa *et al.*, 1988, 1989). For this reason we chose DOX which is comparatively effective on the hepatoma. Some discrepancies exist between the results of our *in vitro* and *in vivo* experiments, which we believe can be explained by considering the time required for DOX to elute into the blood stream around the implantation area. Once the DOX began to elute, it displayed a stable slow release pattern compared with other slow releasing agents. Our study on rabbits showed that the DOX concentrations in serum and organs other than the implanted area were always below a measurable level and the high dose experiment on mice with a tumour did not demonstrate any acute toxicity.

The anticancer effect of DOX-HAP on S-180 depended on the dosage, however, tumour growth inhibition was offset by high mortality due to acute toxicity in high doses in i.t. groups and i.p. groups. Pathological examination revealed an increase of tumour-necrosis around the implantation area and the appearance of necrotic tissue around vessels until 7 days after the procedure. This led us to believe that DOX-HAP produced an anticancer effect not only around the implantation area, but eluted free DOX in the blood stream resulting in the expansion of the necrotic area.

We believe that DOX-HAP implantation therapy is an effective treatment with an efficient drug delivery system. We also discover that careful choice of implantation area and joint use of other therapies, such as embolisation therapy and percutaneous ethanol injection, are required to increase the anti-tumour effect.

### References

AUDISIO, R.A., DOCI, R., MAZZAFERRO, V., BELLEGOTTI, L., TOMMASINI, M., MONTALTO, F., MARCHANO, A., PIVA, A., DEFAZIO, C., DAMASCELLI, B., GENNARI, L. & VANTHIEI, D.H. (1990). Hepatic arterial embolization with microencapsulated mitomycin C for unresectable hepatocallular carcinoma in cirrhosis. *Cancer*, **66**, 228.

BIERMAN, H.R., BYRON, R.L., MILLER, E.R. & SHIMKIN, M.B. (1950). Effects of intra-arterial administration of nitrogen mustard. *Am. J. Med.*, **8**, 535.

DOPPMAN, J.L. CHIRO, G. & OMMAYA, A. (1968). Obliteration of spinal cord arteriovenous malformation by percutaneous embolization. *Lancet*, **1**, 477.

GOLDSTEIN, H.M., WALLACE, S., ANDERSON, J.H., BREE, R.L. & GIANTURCO, C. (1976). Transcatheter occlusion of abdominal tumors. *Radiology*, **120**, 539–545.

GREGORIADIS, G. (1977). Targeting of drugs. *Nature*, **265**, 407–410.

HAGIWARA, A., TAKAHASHI, T., LEE, R., UEDA, T., TAKEDA, M. & ITOH, T. (1987). Chemotherapy for carcinomatous peritonitis and pleuritis with MMC-CH, mitomycin C adsorbed on activated carbon particles. *Cancer*, **59**, 245–251.

HASHIDA, M., KATO, A., KOJIMA, T., MURANISHI, S., SAZAKI, H., TANIGAWA, N., SATOMURA, K. & HIKASA, Y. (1981). Antitumor activity of mitomycin C dextran conjugate against various murine tumors. *GANN*, **72**, 226–234.

KAPLAN, F.L. & MEIER, P. (1958). Nonparametric estimation from incomplete observation. *J. Am. Stat. Assoc.*, **53**, 457–481.

KATO, T., NEMOTO, R., MORI, H. & KUMAGAI, I. (1980). Sustained-release properties of microencapsulated mitomycin C with ethylcellulose infused into the renal artery of the dog. *Cancer*, **46**, 14.

KATO, T. (1983). Encapsulated drugs in targeted cancer therapy. In *Controlled Drug Delivery*, Bruck, S.D. (ed.), pp. 190–240, CRC Press: Florida.

KATO, T., TAMAKAWA, Y. & UNNO, K. (1984). Chemoembolization with mitomycin C microcapsules for hepatoma. *Jpn. J. Soc. Cancer Ther.*, **11**, 798–805.

KLOPP, C.T., ALFORD, T.C., BATEMAN, J., BERRY, G.N. & WINSHIP, T. (1950). Fractionated intra-arterial cancer chemotherapy with methyl bis amine hydrochloride; a preliminary report. *Ann. Surg.*, **132**, 811–832.

KONNO, T., TASHIRO, S. & MAEDA, H. (1983). Treatment of hepatoma with intra-arterial administration of an oily anticancer agent. *Jpn. J. Soc. Cancer Ther.*, **10**, 351–357.

KONNO, T., MAEDA, H., IWAI, K., MAKI, S., TASHIRO, S., UCHIDA, M. & MIYAUCHI, Y. (1984). Selective targeting of anticancer drug and simultaneous image enhancement in solid tumors by arterially administered lipid contrast medium. *Cancer*, **54**, 2367–2374.

NAKAMURA, H., TANAKA, T., HORI, S., YOSHIOKA, H., KURODA, C., OKAMURA, J. & SAKURAI, M. (1983). Transcatheter embolization of hepatocellular carcinoma: assessment of efficacy in cases of resection following embolization. *Radiology*, **147**, 401–405.

OZAWA, S., SUGIYAMA, Y., MITSUHASHI, Y., KOBAYASHI, T. & INABA, M. (1988). Cell killing action of cell cycle phase-non-specific antitumor agents is dependent on concentration-time product. *Cancer Chemother. Pharmacol.*, **21**, 185–190.

OZAWA, S., SUGIYAMA, Y., MITSUHASHI, J. & INABA, M. (1989). Kinetic analysis of cell killing effect induced by cytosin arabinoside and cisplatin in relation to cell cycle phase specificity in human colon cancer and chinese hamster cells. *Cancer Res.*, **49**, 3823–3828.

SAITO, A., TAKASAKI, K., WATAHO, T., AKIMOTO, S., OBATA, H., KOBAYASHI, S. & TSURU, S. (1987). Therapy for small hepatocellular carcinoma using intratumoral injection of apatite-adriamycin solution. *Acta. Hepato. Jpn.*, **24**, 1459.

SEKI, T., NONAKA, T., KUBOTA, Y., MIZUNO, T. & SAMESHIMA, Y. (1989). Ultrasonically guided percutaneous ethanol injection therapy for hepatocellular carcinoma. *Am. J. Gastroenterol.*, **84**, 1400–1407.

SEKI, T., KUBOTA, Y., KUNIEDA, K., KANOU, Y., SATO, M., SHIRO, T., MIZUNO, T., SHIOZAKI, Y. & INOUE, K. (1991). Ultrasonically guided percutaneous ethanol injection therapy for large size hepatocellular carcinoma. *Acta. Hepatol. Jpn.*, **32**, 613–617.

SEMPLE, J.E. (1948). Papillomata of bladder treated with podophyllin; preliminary report. *Br. Med. J.*, **1**, 1235–1237.

SUGIURA, N., TAKARA, K., OTO, M., OKUDA, K. & HIROOKA, N. (1983). Therapy of small hepatocellular carcinoma by ethanol injection ultrasound imaging. *Acta. Hepatol. Jpn.*, **24**, 920.

TAKAHASHI, T., MIZUNO, M., FUJITA, Y., UEDA, S., NISHIDA, B. & MAJIMA, S. (1973). Increased concentration of anticancer agents in regional lymph nodes by fat emulsions, with special reference to chemotherapy of metastasis. *Gann*, **64**, 345–350.

UCHIDA, A., NADE, S., MCCARTNEY, E. & CHING, W. (1985). Bone ingrowth into three different porous ceramics implanted into the tibia of rats and rabbits. *J. Ort. Hp. Res.*, **3**, 65–77.

UCHIDA, A., ARAKI, N. & SHINTO, Y. (1989). Biocompatibility of calcium hydroxyapatite ceramic. *J. Joint Surg.*, **8**, 1755–1760.

URANO, M. & KAHN, J. (1987). Some practical questions in the tumor regrowth assay. In *Rodent Tumor Models in Experiment Cancer Therapy*. Kallman, R. (ed.), pp. 122–127, Pergtamon Press: Elmsford, NY.

WIDDER, K.J., SENYEI, A.E. & RANNEY, D.F. (1979). Magnetically responsive microspheres and other carriers for the biophysical targeting of antitumor agents. *Chemother.*, **16**, 213–217.

YAMADA, R., SATO, M., KAWABATA, M., NAKATSUKA, H., NAKAMURA, K. & TAKASHIMA, S. (1983). Hepatic artery embolization in 120 patients with unresectable hepatoma. *Radiology*, **148**, 397–401.